# Walking with head-mounted virtual and augmented reality devices: Effects on position control and gait biomechanics

**DOI:** 10.1371/journal.pone.0225972

**Published:** 2019-12-04

**Authors:** Zoe Y. S. Chan, Aislinn J. C. MacPhail, Ivan P. H. Au, Janet H. Zhang, Ben M. F. Lam, Reed Ferber, Roy T. H. Cheung

**Affiliations:** 1 Gait & Motion Analysis Lab, Department of Rehabilitation Sciences, The Hong Kong Polytechnic University, Hung Hom Bay, Hong Kong S.A.R; 2 Running Injury Clinic, University of Calgary, Calgary, Canada; 3 Faculties of Kinesiology, Nursing, and Cumming School of Medicine, University of Calgary, Calgary, Canada; University of Memphis, UNITED STATES

## Abstract

What was once a science fiction fantasy, virtual reality (VR) technology has evolved and come a long way. Together with augmented reality (AR) technology, these simulations of an alternative environment have been incorporated into rehabilitation treatments. The introduction of head-mounted displays has made VR/AR devices more intuitive and compact, and no longer limited to upper-limb rehabilitation. However, there is still limited evidence supporting the use of VR and AR technology during locomotion, especially regarding the safety and efficacy relating to walking biomechanics. Therefore, the objective of this study is to explore the limitations of such technology through gait analysis. In this study, thirteen participants walked on a treadmill in normal, virtual and augmented versions of the laboratory environment. A series of spatiotemporal parameters and lower-limb joint angles were compared between conditions. The center of pressure (CoP) ellipse area (95% confidence ellipse) was significantly different between conditions (*p* = 0.002). Pairwise comparisons indicated a significantly greater CoP ellipse area for both the AR (*p* = 0.002) and VR (*p* = 0.005) conditions when compared to the normal laboratory condition. Furthermore, there was a significant difference in stride length (*p*<0.001) and cadence (*p*<0.001) between conditions. No statistically significant difference was found in the hip, knee and ankle joint kinematics between the three conditions (*p*>0.082), except for maximum ankle plantarflexion (*p* = 0.001). These differences in CoP ellipse area indicate that users of head-mounted VR/AR devices had difficulty maintaining a stable position on the treadmill. Also, differences in the gait parameters suggest that users walked with an unusual gait pattern which could potentially affect the effectiveness of gait rehabilitation treatments. Based on these results, position guidance in the form of feedback and the use of specialized treadmills should be considered when using head-mounted VR/AR devices.

## Introduction

Over the past two decades, the application of virtual reality (VR) technology in a healthcare setting has become increasingly popular. It has been incorporated into clinical practices such as in the rehabilitation of stroke survivors, as well as patients with cerebral palsy and multiple sclerosis [1–3]. There is ample evidence suggesting that VR-based rehabilitation facilitates upper limb motion [4] and dynamic balance [5] among stroke survivors. More recently, research groups have also investigated the use of VR in dynamic situations (i.e. treadmill walking), aiming to improve balance and facilitate gait recovery [6–9].

In current clinical practice, gait retraining often includes treadmill training under the supervision of practitioners or through provision of real-time biofeedback. It is a widely adopted technique that aims to permanently correct faulty gait patterns and has been found to be effective in both walking and running gait modifications [10–12]. For example, a recently published randomized controlled trial showed that gait retraining was an effective intervention for reduction of knee loading and also improved symptoms among patients with early knee osteoarthritis [10]. Incorporation of VR technology into conventional gait retraining has the potential to further enhance training outcomes. VR allows users to actively interact with a simulated environment in real-time and offers the opportunity to practice skills acquired in the virtual environments to everyday life [13]. VR-based gait retraining has the potential to facilitate implicit learning, enhance variety, and actively engage the patient during training. These attributes are crucial in the optimization of motor learning and could maximize the training effect [14].

Walking is normally an automatic process. It has been suggested that conscious modification to walking patterns could affect gait retraining adaptations [15]. A previous study found that subjects who trained with distraction were able to retain the training effect longer than the group who focused on correction [15]. VR-based retraining could include different tasks and games while the patients modify their gait pattern as it could help patients to maintain focus and promote implicit motor learning. Moreover, the training environment, feedback type and level of difficulty of tasks can be manipulated within the VR environment relatively effortlessly for the clinician, as compared to conventional gait retraining. Variation in training has been shown to promote a more robust motor pattern and favor adaptation [16,17]. Moreover, motivation and adherence among patients can also be improved with more variation and an adjustable level of difficulty provided in the VR-based training [18]. Stroke survivors were previously found to be more actively engaged in a VR-based training than a conventional task-oriented intervention to improve motor function [19]. The training environment can be designed to simulate real-life activities and include task-specific training and a natural experience can be achieved through immersive VR devices, such as using a head-mounted display (HMD) [20]. Studies have supported task-specific motor skill training with VR in helping to drive neuroplasticity in individuals with progressive neurodegenerative disorder [4,21].

Although multiple studies have reported positive results of gait retraining using VR among various patient groups within the lab [1,5,22,23], there is still little understanding of the limitations and challenges for using VR technology clinically. One overriding concern for using VR technology in clinical applications, especially an HMD, is safety. The user may not be able to recognize his/her own body position when using an immersive VR device, which could result in physical injuries, particularly if the user fails to stay within the boundaries of the treadmill. Suspension devices (i.e. an over-head harness) have been used for protection during VR-based gait rehabilitation [8], and a recent study showed that both young and older adults were able to use HMD during walking without adverse effects [21]. However, the limit of VR technology on safety was not quantified or discussed. Recent technological advances in both the hardware and software of HMD might allow for safer use. However, there is still a need for evidence-based support and quantifiable data, which could help with practical considerations among VR applications in a clinical setting.

Another concern for gait rehabilitation would be the regularity and quality of gait. Through studying spatiotemporal gait parameters, some studies have reported that walking in a projected VR environment can induce gait instability even in healthy participants [24,25]. Nowadays, VR-based gait retraining using HMD focuses primarily on gait restoration after stroke [8]; the changes in natural gait due to the use of HMD may not be clinically significant. However, it is crucial for particular patient groups undergoing gait modification to maintain a certain level of regularity in their gait pattern. For instance, knee loading can be affected by spatiotemporal parameters such as cadence and step length [26] and VR was previously found to alter such parameters in an over-ground setting [24]. The treatment effect of gait retraining in reducing knee loading would likely be affected if the patient’s baseline walking gait was already altered by the use of HMD or other VR devices. The aforementioned studies did not quantify the changes in walking biomechanics when using a HMD, therefore, this study aimed to identify gait parameters that were affected by the use of HMD.

An alternative to VR is Augmented Reality (AR), which does not fully immerse the user in a simulated environment but includes virtual elements that are superimposed on a real-world view [27]. For example, external cues on foot placement could be overlaid on to the walking surface in order to facilitate gait adjustments [28,29]. The addition of feedback in AR-based gait retraining allows for variations in training and could enhance the gait retraining effect. Yet, there is also a lack of understanding of the limitation of using AR devices. Therefore, this study also aimed to examine the biomechanical changes induced by the HMD within an AR setting.

This study was designed to assess whether the use of commercially available HMD in VR and AR settings were suitable for clinical gait retraining. Specifically, the aim was to quantify the limitations of current VR and AR technology based on two practical concerns for clinical applications: 1) safety: the ability of the user to maintain a relatively stable position within the treadmill and 2) natural gait patterns: deviation of walking biomechanics from that of normal-treadmill walking. We hypothesized that there would be variations in the control of body position relative to the treadmill between both VR and AR conditions when compared with normal-treadmill walking. Also, based on altered gait biomechanics reported with the use of HMD in an over-ground setting [24], we hypothesized there would be variation in the spatiotemporal and joint kinematic measures while walking in VR and AR conditions, when compared with normal-treadmill walking.

## Materials and methods

### Participants

A total of 13 participants (7 females, 6 males; age = 24.6 ± 4.5 years; weight = 63.1 ± 14.5 kg; height = 1.68 ± 0.11 m) were recruited for this study through convenient sampling, which is a comparable sample size to previous studies [30–32]. Participants were free of any musculoskeletal, neurological, neuromuscular or cardiovascular pathology that might hinder walking. The experimental procedures were reviewed and approved by the Departmental Research Committee of the department of Rehabilitation Sciences, The Hong Kong Polytechnic University (Ref.: HSEARS20161018001) and written informed consent was obtained from all participants prior to the experiment.

### Experimental procedures

Participants were asked to walk at a self-selected pace for four minutes to allow for treadmill adaptation prior to data collection [33]. Anthropometric data, including leg length, knee width and ankle width [34–36], were recorded and 39 reflective markers were affixed to specific bony landmarks based on the Vicon Plug-in-Gait® full body model [34]. The marker model was previously established for the measurement of lower-limb kinematics [35]. This study was designed to assess HMD in VR and AR settings using a commercially available model within a typical clinical setting. Thus, the conditions were designed to be simple and without the use of additional lab equipment. All walking trials were conducted on a dual-belt instrumented treadmill (Force-sensing tandem treadmill, AMTI, Watertown, MA, USA; length x width = 1.2 x 0.6 m). Participants wore their own usual shoes and walked under different conditions at 3.0 km/h (0.83 m/s) for three minutes each. The three conditions were Control, VR and AR, details were as follows:

Control: Treadmill walking without the HMD;

Virtual reality (VR): Immersive 360° panoramic image of the laboratory captured by the Samsung Gear 360 Cam (Samsung, Seoul, South Korea), set up instructions and image file used are provided in the supporting information (S1 File and S1 Fig).

Augmented reality (AR): Real-time display through the rear camera of the HMD, set up instructions are provided in the supporting information (S2 File).

For the AR and VR conditions, participants wore a head-mounted VR device (Samsung Gear VR SM-R322 and Samsung Galaxy S7, Samsung, Seoul, South Korea; width x height x depth: 201.93 x 92.71 x 116.33 mm). The immersive VR/AR environment within this study refers to the panoramic display in a first-person perspective with complete visual obstruction to the real-world environment. The HMD used in this study weighs a total of 470 g, which is comparable to typical commercial HMD models (HTC VIVE Pro: 555 g [37] and Oculus Rift DK2: 440 g [38]). Adjustments to the device were made for fit, focus, and orientation for each participant. Participant’s comfort was confirmed through subjective reporting before the beginning of each walking trial.

**Fig 1:**
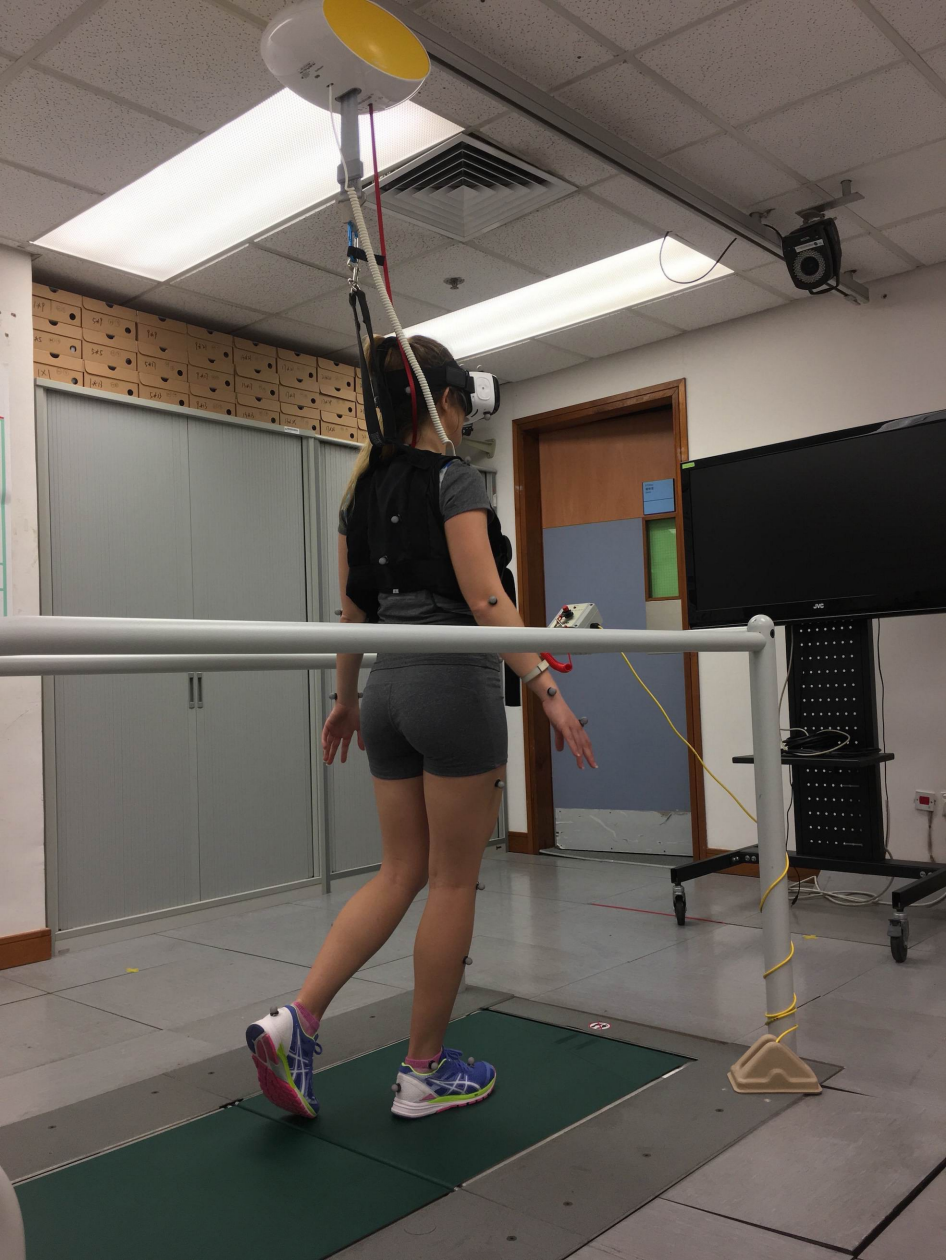
A photograph to illustrate the experimental setup. For condition AR and VR, the participant wore a head-mounted VR device. The participant was protected by an overhead safety harness system. Reflective markers and motion cameras were employed to collect gait biomechanics during the walking trials.

The test sequence was randomized using a web-based software (www.randomizer.org). To ensure safety, participants were supported by an overhead safety harness providing 0% bodyweight support. The experimental setup is indicated in Fig 1. The individual in Fig 1 of this manuscript has given written informed consent (as outlined in PLOS consent form) to publish the photograph.

Ground reaction force and coordinates of the center of pressure (CoP) were sampled through the instrumented treadmill at 1,000 Hz. Marker trajectories were sampled at 200 Hz using an 8-camera motion capture system (Vicon, Oxford Metrics Group, UK). The instrumented treadmill and motion capture system were synchronized and were set for data collection for three minutes after the treadmill reached the testing speed.

### Data processing

A threshold of 10 N was used to define initial contact and toe-off based on the vertical component of the ground reaction force data [39]. Stride length was calculated as the product of the treadmill speed and the time between successive initial contacts of the same foot [40]. Cadence was calculated as the number of footfalls per minute. The CoP 95% confidence ellipse area was calculated using a tailored MATLAB program (The MathWorks, Inc, Natick, MA, USA) according to the method established by Schubert and Kirchner [41]. The mediolateral (perpendicular to the direction of travel) and anteroposterior (along the direction of travel) boundary of the CoP ellipse were also obtained (S2 Fig).

The raw marker trajectories were low-pass filtered at 12 Hz with a fourth order Butterworth filter [42]. Lower-limb joint angles in the sagittal and coronal plane were computed with the Dynamic Plug-in pipeline based on the validated subject-specific model built using the collected anthropometry data, including leg length, knee width and ankle width [34–36]. The maximum and minimum values of the hip, knee and ankle joint kinematics were obtained for each gait cycle and averaged across all gait cycles within the data collection period of each condition.

### Statistical analysis

One-way repeated measures ANOVAs were used to compare CoP ellipse area, mediolateral and anteroposterior boundaries of the CoP ellipse, stride length, cadence, and lower-limb joint angles between the three conditions. Pairwise comparisons with Bonferroni correction were conducted when necessary. Cohen’s *d* was calculated to evaluate the effect size. Statistical tests were performed using SPSS Version 22 (Chicago, IL, USA).

## Results

**Fig 2:**
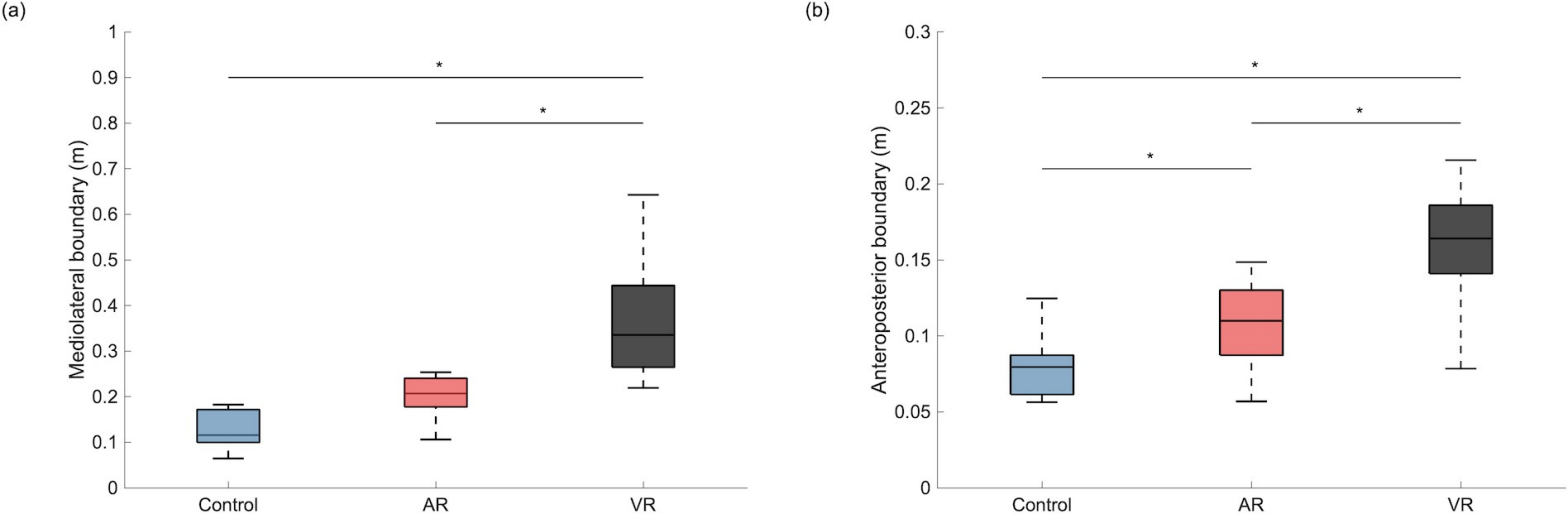
**The group average and standard deviation of the (a) mediolateral and (b) anteroposterior boundary of the center of pressure ellipse under different walking conditions.** AR: augmented reality; VR: virtual reality. Asterisk (*) denotes p<0.05 in the corresponding pairwise comparison.

Individual CoP ellipses were plotted for each condition (S3 Fig). The mean and standard deviation (SD) are presented in Table 1. One-way repeated measures ANOVA indicated that the mean CoP ellipse area was significantly different between conditions (F = 12.55, *p* = 0.002). Pairwise comparisons indicated a significantly greater CoP ellipse area for both the AR (*p* = 0.002, Cohen’s *d* = 1.79) and VR (*p* = 0.005, Cohen’s *d* = 1.53) conditions when compared to Control. Moreover, both the mediolateral (F = 16.17, *p*<0.001) and anteroposterior (F = 39.60, *p*<0.001) boundaries of the CoP ellipse were found to be significantly different between conditions. Pairwise comparisons indicated that the mediolateral boundary (Fig 2A) was significantly greater in the VR condition when compared to Control (*p* = 0.001, Cohen’s *d* = 2.07) and AR condition (*p* = 0.008, Cohen’s *d* = 1.18). Similarly, the anteroposterior boundary (Fig 2B) was also found to be significantly greater in the VR condition than both Control (*p*<0.001, Cohen’s *d* = 2.30) and AR conditions (*p*<0.001, Cohen’s *d* = 1.72). Apart from that, the anteroposterior boundary in the AR condition was also significantly greater than Control (*p* = 0.049, Cohen’s *d* = 0.81).

**Table 1:** Stride length, cadence and center of pressure ellipse area of different walking conditions.

	Control	Augmented reality	Virtual reality	ANOVA *P-*value
CoP ellipse area, cm^2^	124.28 ± 86.24^b^^,^^c^	433.78 ± 229.27^a^	934.14 ± 745.09^a^	0.002*
Stride length, m	1.03 ± 0.05^b^^,^^c^	0.98 ± 0.07^a^	0.98 ± 0.06^a^	< 0.001*
Cadence, steps/min	96.86 ± 4.68^b^^,^^c^	102.41 ± 7.90^a^	102.73 ± 6.59^a^	< 0.001*

* One-way repeated measures ANOVA: *P*<0.05.

a: significantly different from Control.

b: significantly different from Augmented reality.

c: significantly different from Virtual reality.

Individual mean values for spatiotemporal and kinematic parameters under each condition are presented in S3 File. Table 1 also presents the mean and SD of the temporospatial parameters. Results of the one-way repeated measures ANOVAs demonstrated that both stride length (F = 12.16, *p*<0.001) and cadence (F = 10.89, *p*<0.001) were significantly different between conditions. Pairwise comparisons indicated that the stride length in the AR (*p* = 0.002, Cohen’s *d* = 0.85) and VR (*p* = 0.001, Cohen’s *d* = 1.03) conditions were significantly shorter when compared to Control, while cadence was higher in the AR (*p* = 0.004, Cohen’s *d* = 0.86) and VR (*p* = 0.001, Cohen’s *d* = 1.03) conditions when compared to Control.

Table 2 presents the mean and SD of the hip, knee and ankle joint in both the sagittal and coronal plane under different conditions. One-way repeated measures ANOVAs demonstrated no significant differences in the hip and knee joints (*p*>0.082). The maximum ankle plantarflexion angle was significantly different between conditions (F = 12.05, *p* = 0.001). Pairwise comparisons indicated a significantly smaller plantarflexion angle for both the AR (*p* = 0.001, Cohen’s *d* = 0.46) and VR (*p* = 0.009, Cohen’s *d* = 0.67) conditions when compared to Control.

**Table 2:** The maximum and minimum lower-limb joint angles measured during different walking conditions.

	Control	Augmented reality	Virtual reality	ANOVA *P*-value
**Hip**				
Flexion; maximum, °	26.22 ± 5.38	25.29 ± 6.72	26.40 ± 5.91	0.513
Extension; maximum, °	10.19 ± 4.63	10.62 ± 6.08	7.91 ± 7.25	0.083
Adduction; maximum, °	6.11 ± 3.96	5.45 ± 3.22	4.76 ± 3.25	0.664
Abduction; maximum, °	8.53 ± 2.90	8.37 ± 3.04	8.82 ± 3.09	0.090
**Knee**				
Flexion; maximum, °	62.70 ± 8.99	61.79 ± 9.68	62.10 ± 9.88	0.360
Flexion; minimum, °	1.23 ± 6.20	2.23 ± 5.63	3.33 ± 5.89	0.082
Adduction; maximum, °	15.84 ± 16.18	14.86 ± 15.10	13.85 ± 4.58	0.527
Abduction; maximum, °	9.40 ± 8.75	9.07 ± 7.20	10.74 ± 9.74	0.326
**Ankle**				
Dorsiflexion; maximum, °	14.42 ± 5.17	14.44 ± 4.32	16.32 ± 7.32	0.154
Plantarflexion; maximum, °	17.53 ± 6.78^b^^,^^c^	14.63 ± 5.68^a^	13.59 ± 4.57^a^	0.001*
Inversion; maximum, °	0.41 ± 3.05	1.10 ± 3.79	0.93 ± 3.42	0.218
Eversion; maximum, °	7.15 ± 4.91	6.73 ± 4.72	7.28 ± 4.89	0.537

* One-way repeated measures ANOVA: *P*<0.05.

a: significantly different from Control.

b: significantly different from Augmented reality.

c: significantly different from Virtual reality.

## Discussion

The purpose of this study was to assess the use of an HMD in VR and AR settings based on the ability of the user to maintain a relatively stable position on the treadmill and the effect on walking biomechanics compared to that of normal-treadmill walking. The CoP ellipse area was significantly greater in both the AR and VR conditions compared to Control and stride length and cadence in the AR and VR conditions were also found to be significantly different from Control. Regarding lower-limb joint kinematics, no significant differences were found between conditions in the hip and knee joint angles. However, the maximum ankle plantarflexion angle was found to be smaller than Control in both the AR and VR conditions.

In this study, the CoP 95% confidence ellipse was computed to indicate the position of the participant within the treadmill during the data collection period. The CoP ellipse area could reflect the ability of the participant to maintain a stable direction during locomotion and also their sense of orientation under different conditions. The high variability observed in the VR and AR conditions reflects a discrepancy among individuals responding to immersive VR and AR using an HMD (S3 Fig and S3 File). Overall, participants adopted different strategies in maintaining their position within the center of the treadmill when visual information provided was different from reality. In general, a larger CoP ellipse area was observed in the AR and VR conditions when compared to Control. These results suggest that an HMD with AR or VR settings affects the participant’s sense of orientation relative to the treadmill. Future studies could look into head movements or the attention of focus while using the HMD device to further understand the between-participant differences. Moreover, the dimension of the walking surface should be considered and additional guidance may be necessary for using AR and VR devices for clinical gait retraining. In order to better understand the difference in the shifting of CoP perpendicular to and along the direction of travel, the change in mediolateral and anteroposterior boundaries of the CoP ellipse were isolated respectively for further comparison between conditions.

The mediolateral boundary of the CoP ellipse was significantly larger in the VR condition when compared to Control. Interestingly, such a difference was not observed between the AR and Control and participants were better able to control their mediolateral sway under the AR than under the VR condition. Visual information helps in the control of locomotion [43] and humans rely on egocentric direction based on visual information, which allows movement in the perceived direction of a target [44]. The AR provides real-time visual information of a person relative to the laboratory environment, for example, when the participant shifted towards one’s right side, the objects within the visual field would move in the opposite direction (i.e. left). The visual information provided through the AR setting allows the participants to recognize their relative position within the treadmill and the laboratory, and therefore able to adjust their heading direction accordingly. However, the VR setting used within this study was sensitive to the rotation (pitch, yaw and roll) but not the translation of the participant and the panoramic VR environment was created from a single viewpoint. When rotation was detected by the HMD, the display within the visual field would change correspondingly. For example, when the participant looks towards the right by turning the head, the right sidewall of the laboratory would be visible within the display. However, when the participate takes a step towards the right, the display would remain unaltered. Participants were unaware of any lateral shift based on the visual display which might have affected the participants’ ability to maintain their body position within a small mediolateral boundary.

The anteroposterior boundary of the CoP ellipse was significantly greater in both the AR and VR conditions compared to Control, despite the shorter stride lengths found in both treatment conditions. A reasonable explanation for such changes would be the anteroposterior shifting in the participant’s body position relative to the treadmill. The increase in the anteroposterior boundary might also be explained by the lack of depth perception in both treatment conditions. In reality, an object is projected onto the left and right eye at a slightly different angle, which provides information for the brain to perceive depth. The real-time visual display in AR setting and the panoramic image in the VR setting were both monoscopic, meaning that the same image was being displayed to both eyes. The monoscopic display was perceived as a flat surface, like a painting, and therefore depth information was not available. Past research has found that distances tend to be under-perceived in virtual environments [45,46] and the reduction in stride length might also be explained by the mitigated depth perception in the treatment conditions. Indeed, the anteroposterior boundary was still significantly smaller in AR than VR and the change in position and size of objects within the visual display, which was available under the AR setting, might compensate for the lack of depth perception to a certain extent. These results suggest that AR provided more information for the user to maintain their position in both the mediolateral and anteroposterior direction. Regardless, in this study, both the VR and AR settings adopted monoscopic displays. While this setting retained a certain level of realism, the level of immersion may not have been adequate for participants to maintain their body position, especially under the VR setting. Position information and additional guidance should be considered for users of VR devices to maintain their position. Also, more advanced VR device settings, which use stereoscopic images created with multiple lens or computer-generated three-dimensional environments and therefore preserve depth information, should be considered for gait rehabilitation on a treadmill.

Regarding walking biomechanics, differences were observed between the VR and AR conditions when compared with Control. Reduced stride length and increased cadence are examples of gait modifications commonly utilized to increase stability. In general, these gait adjustments are adopted to reduce perturbations to the body and therefore reduce fall risk [47]. Specifically, a shorter stride length minimizes the forward movement of the CoP beyond the base of support provided by the stance foot, thus increasing stability [47]. In a previous study, a group of young adults demonstrated reduced stride length and increased cadence when blindfolded [48], reflecting a more cautious walking strategy when visual feedback was removed. However, in the present study, walking speed was kept constant for all walking conditions and the reduction in stride length was likely coupled with the reported increase in cadence. These changes indicate that participants adopted a cautious walking strategy under unfamiliar walking conditions using the VR device.

Past studies have demonstrated that the ankle plantorflexors, such as the gastrocnemius and soleus, contribute to bodyweight support and propulsion [49,50]. The maximum ankle plantarflexion angle during push-off was smaller during walking in both the AR and VR conditions than in Control. This result suggests a reduction in the participant’s willingness to propel forward with the same confidence as in the regular treadmill walking, which is also supported by the reduction in stride length and increase in cadence. Together with the changes in spatiotemporal parameters, the reduction in ankle plantarflexion at toe-off suggests that participants modify their gait pattern under the AR and VR conditions, with the ankle being most sensitive among the lower limb joints. Additionally, an unnatural gait pattern could impact the effectiveness of gait retraining, and should therefore not be ignored when designing gait retraining protocols that incorporate VR/AR technology.

There are VR devices and software specially designed for gait rehabilitation, which may also require the concurrent use of motion tracking devices or specially designed treadmills. However, such devices may not be readily available inside a rehabilitation clinic. The HMD used in this study was a lightweight, commercially available model and the setup was simple and did not require specific technical modifications. However, the conditions within this study cannot represent all HMD applications in gait rehabilitation and the aim of this study was to identify areas of improvement for VR and AR treatment using HMD. The large CoP area and boundaries suggest that the use of an HMD on a conventional treadmill may be rather dangerous if guidance on position is unavailable, and therefore, hardware and software enhancement might be necessary. Solutions like using IMU-based position and orientation tracking [51] or an omni-directional treadmill (Indinadeck, Rocklin, California, USA) might help to tackle the problem of using a similar HMD device as used in the current study within a clinical setting. Another potential solution could be adopting a computer-generated optic flow [8] that matches with the treadmill speed, which could help improve the congruency between the users’ proprioceptive sensory information and the visual feedback provided in the VR devices.

Moreover, our findings on altered walking biomechanics provides an example of the limitations for VR and AR applications. A natural gait pattern is the underpinning for the effectiveness of gait retraining, and while the disruption of gait patterns may be inconsistent across various HMD devices and under different settings (VR *vs*. AR, motorized *vs*. non-motorized treadmill, HMD *vs*. projector-based VR), the naturalness of gait should be measured for each specific application and the length of the adaptation period likely depends on the setting as well. For example, some previous studies using HMD in a VR setting did not report the length of an adaptation period [9,52], with only one reporting a 10 minute adaptation [8]. There is no consensus within the literature regarding the amount of time needed for adaptation, and it is likely different in various settings and patient groups. Results of our study support the need for an application-specific adaption period to be determined if effectiveness of the treatment is likely affected by altered gait patterns. Moreover, walking biomechanics, especially spatiotemporal parameters and ankle kinematics, should be investigated before considering VR and AR applications for gait retraining.

Results presented in this study should be considered in view of other limitations to the experiment. First, the walking trials were conducted on a motorized treadmill. As well, the direction of travel and the walking speed were fixed, therefore the effect of HMD VR/AR devices on the heading direction, walking speed and gait parameters during over-ground walking remain unknown. However, this study aimed to test the current limitation of VR-based technology on gait rehabilitation, therefore it is appropriate to consider the use of VR on treadmills where gait retraining is usually conducted. Second, the participants of this study consisted of only healthy young adults, which could limit the generalizability of our findings to other populations, such as older users or patient groups. There could also be potential differences in the response of an immersive VR environment including postural instability, motion sickness, increased heart rate, and other autonomic reactions [53,54]. It is therefore suggested that the design of future training protocols should consider repeating a similar experiment on specific patient groups within a clinical setting. Third, the fixed walking speed within this study was slower than the comfortable speed range of healthy young adults (1.05–1.43 m/s) [55], but it was comparable to the reported speed used in a previous gait retraining study (0.86 ± 0.17 m/s) [10]. Finally, only one VR device was tested in this experiment, and therefore future studies should consider other models that support stereoscopic displays and position guidance.

## Conclusions

Through technological advancement, the application of VR to rehabilitation has evolved. This study has explored the limitations of VR and AR applications through a simple setup using an HMD. Results of this study called attention to safety measures and position guidance when using an HMD for gait retraining rehabilitation. Also, potential alterations of gait biomechanics should be taken into consideration when designing VR/AR-based treatment protocols.

## Supporting information

S1 Fig Immersive 360° panoramic image of the laboratory used in condition VR. VR: virtual reality.
(JPG)

S2 Fig Mediolateral (ML) and anteroposterior (AP) boundary of the center of pressure ellipse. (TIFF)

S3 Fig **Individual center of pressure ellipses under the (a) control condition, (b) augmented reality condition and (c) virtual reality condition.** Each color represents a different participant. The same color is used across conditions.
(TIFF)

S1 File Instructions for setting up the virtual reality using S1 Fig. (DOCX)

S2 File Instructions for setting up the augmented reality using the in-built passthrough feature. (DOCX)

S3 File Individual mean value for spatiotemporal and kinematic parameters. AR: augmented reality; VR: virtual reality.
(XLSX)
