# Supplementary figures and images for: Walking with head-mounted virtual and augmented reality devices: Effects on position control and gait biomechanics
Source: PLoS One. 2019 Dec 4;14(12):e0225972. doi: 10.1371/journal.pone.0225972 (PMC6892508; doi:10.1371/journal.pone.0225972)

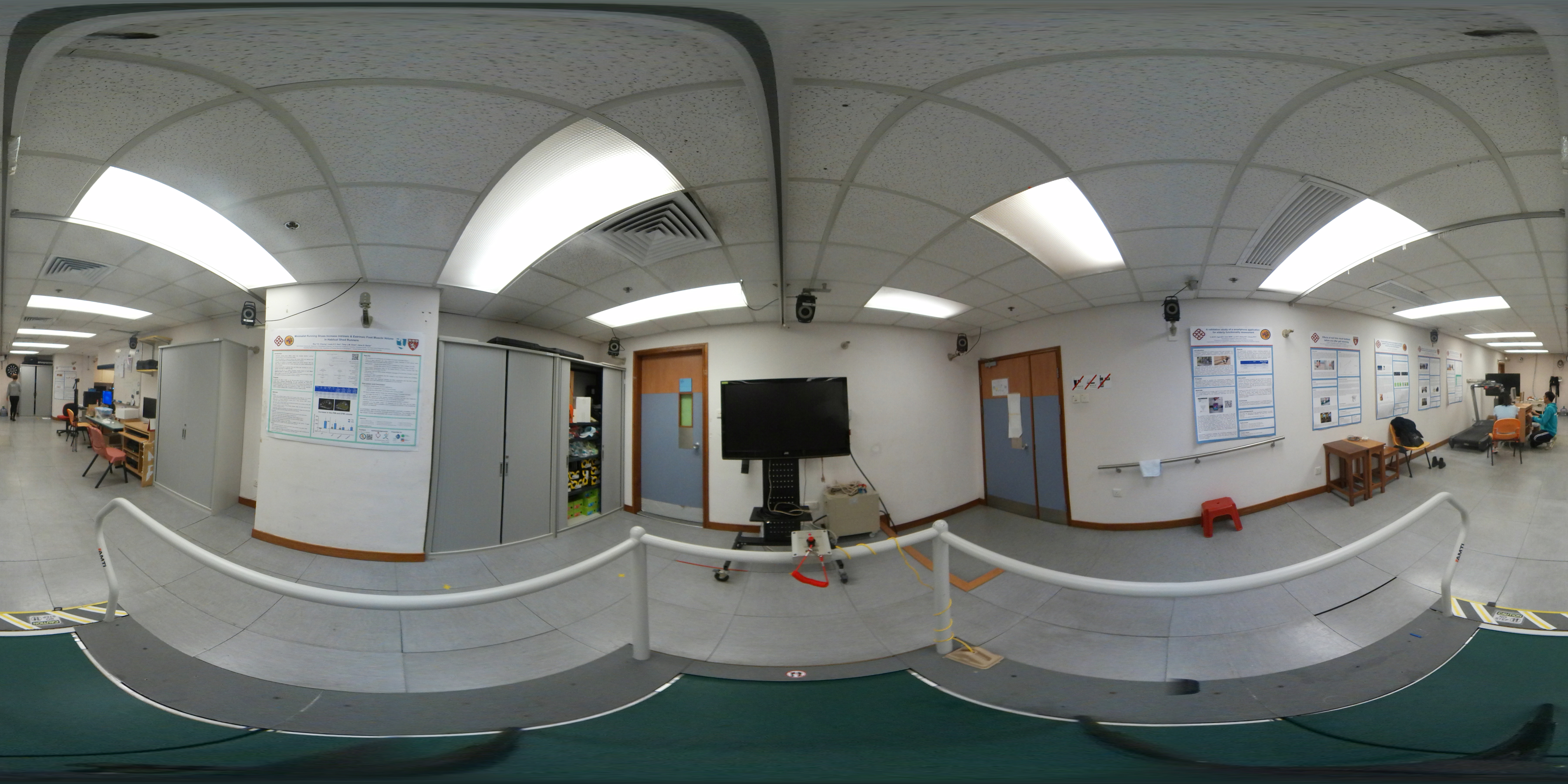

Supplement: S1 Fig — VR: virtual reality. (JPG) [file pone.0225972.s001.jpg]

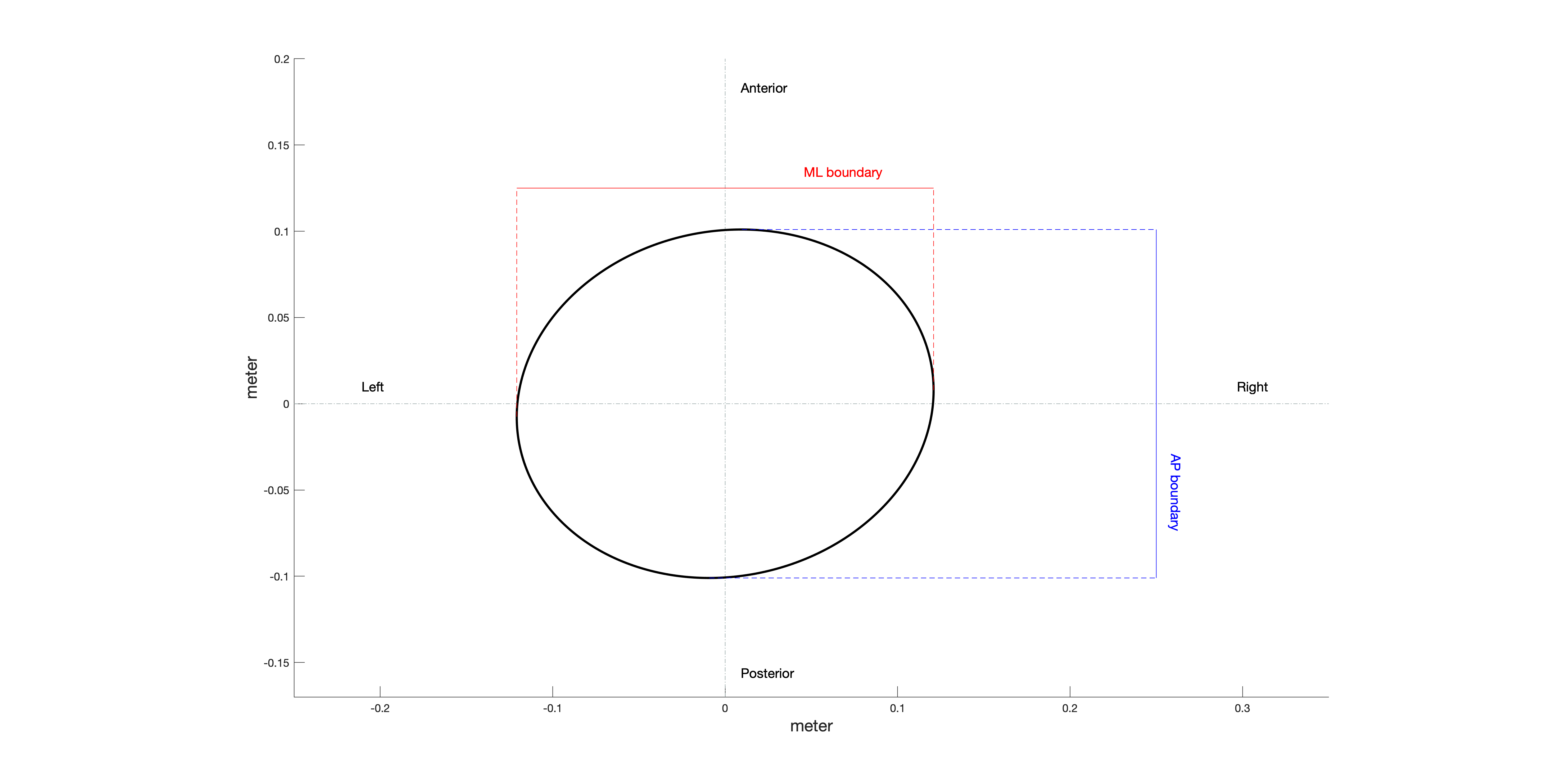

Supplement: S2 Fig — (TIFF) [file pone.0225972.s002.tiff]

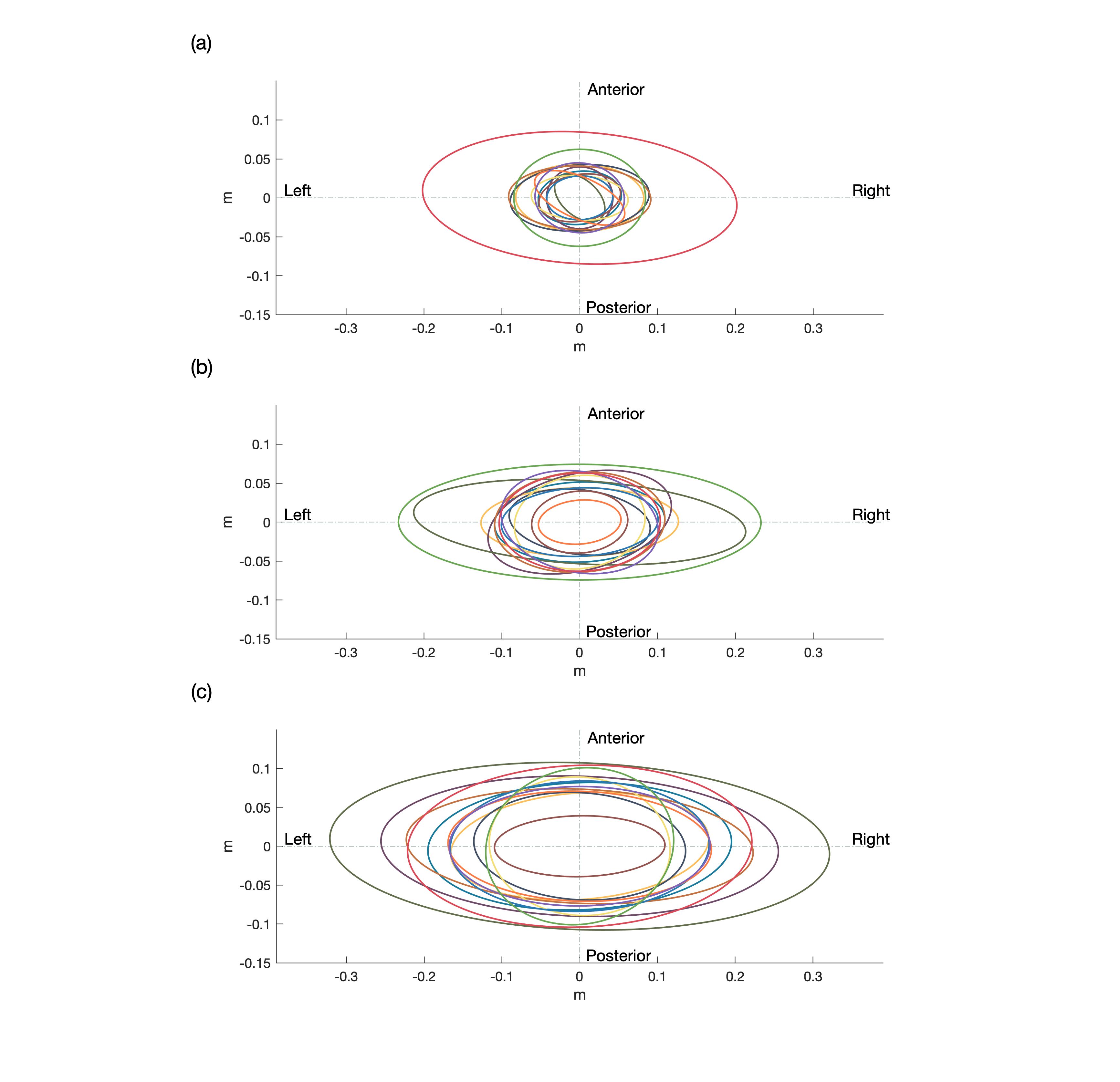

Supplement: S3 Fig — Individual center of pressure ellipses under the (a) control condition, (b) augmented reality condition and (c) virtual reality condition. Each color represents a different participant. The same color is used across conditions. (TIFF) [file pone.0225972.s003.tiff]
