# Supplementary material for: Walking with head-mounted virtual and augmented reality devices: Effects on position control and gait biomechanics
Source: PLoS One. 2019 Dec 4;14(12):e0225972. doi: 10.1371/journal.pone.0225972 (PMC6892508; doi:10.1371/journal.pone.0225972)
Supplement: S1 File — (DOCX) [file pone.0225972.s004.docx]

Instructions for head-mounted display with VR setting

Manufacturer’s instruction:

“Using Oculus 360 Photos on Your Gear VR”

<https://www.samsung.com/us/support/answer/ANS00062990/>

Steps:

1. Save supporting information “S1 Fig.” into the device
2. Launch Oculus Home screen
3. Navigate to Library
4. Select Oculus 360 Photos
5. Under MyPhotos, select S1 Fig.
