# Supplementary material for: Walking with head-mounted virtual and augmented reality devices: Effects on position control and gait biomechanics
Source: PLoS One. 2019 Dec 4;14(12):e0225972. doi: 10.1371/journal.pone.0225972 (PMC6892508; doi:10.1371/journal.pone.0225972)
Supplement: S2 File — (DOCX) [file pone.0225972.s005.docx]

Instructions for head-mounted display with AR setting

Manufacturer’s instruction:

“Passthrough Feature on Gear VR”

https://www.samsung.com/us/support/troubleshooting/TSG01111304/

Description:

The passthrough feature activates the phone's rear camera and projects it onto the screen to show your surroundings while wearing the Gear VR

Steps:

1. Remove the front cover of Gear VR
2. Press and hold the Back key on the Gear VR
3. From the Device tab, select Passthrough
